# A prognostic assessment predicated by blood culture-based bacteria clustering from real-world evidence: Novel strategies and perspectives on prevention and management of sepsis

**DOI:** 10.3389/fmolb.2023.1160146

**Published:** 2023-03-30

**Authors:** Shaokang Xu, Jizhen Cai, Ahmed Doomi, Jian Shi

**Affiliations:** ^1^ Department of Hematology and Critical Care Medicine, The Third Xiangya Hospital, Central South University, Changsha, China; ^2^ Miller School of Medicine, University of Miami, Miami, FL, United States; ^3^ Jackson Memorial Hospital, Miami, FL, United States

**Keywords:** sepsis, prognostic assessment, blood culture, machine learning, bacteria clustering, real-world evidence, severe infection, multiple organ dysfunction syndrome

## Abstract

Sepsis, a syndrome with disturbed host response to severe infection, is a critical health problem worldwide. It is urged to develop and update novel therapeutic strategies for improving the outcome of sepsis. In this study, we demonstrated that different bacteria clustering in sepsis patients may generate differences of prognosis results. We extracted all the sepsis patients from Medical Information Mart for Intensive Care IV 2.0 (MIMIC-IV 2.0) critical care data set according to certain standards and clinical score, a total of 2,339 patients were included in our study. Then we used multiple data analytics and machine learning methods to make all data deeply analyzed and elucidated. The results showed that the types of bacteria infected by patients with different ages, sex and race are different, the types of bacteria infected by patients with different SIRS values and GCS scores of the first day are different, and the severity of patients with different clusters is different, and most importantly, the survival rate of patients with different clusters also has this significant difference. We concluded prognostic assessment predicated by bacteria clustering might be a relatively potentially novel strategies and perspectives on prevention and management for sepsis in the future.

## 1 Introduction

Sepsis is a very serious life-threatening condition that usually happens when the body responds to different kinds of infection and harms the tissues, which may likely progress to septic shock, organ failure and even death (Matot and Sprung, 2001; Riedemann et al., 2003a; Hotchkiss and Karl, 2003). Commonly, when the infection-fighting processes turn on the body, they cause organs to function abnormally and badly (Remick, 2007; Gotts and Matthay, 2016; Jarczak et al., 2021), bacterial infections are one of the indispensable causal factors. Although early treatments with intravenous fluids and symptomatic antibiotics have been proven to have positive therapeutic effects and could improve chances for survival (Riedemann et al., 2003b; Polat et al., 2017; Chen et al., 2019) and a series of novel therapeutic strategies and targets have been found (Shi et al., 2021; Tang et al., 2021; Wang et al., 2021; Li et al., 2022), the prevalence of sepsis is increasing globally with an augment of multidrug-resistant bacteria, viral infection, and the aging population. Thus, it is urged to develop or update therapeutic strategies and real-world evidence for improving the outcome in the treatment of sepsis.

Some of the most frequently isolated bacteria in sepsis are Gram-negative bacteria such as *Escherichia coli* and *Klebsiella pneumoniae* (Ramachandran, 2014). In clinical practice, when treating or evaluating the prognosis of patients with sepsis, after selecting the corresponding drugs, it is less likely to analyze the synergy or competitive inhibition of various bacteria. For example, Gram-negative bacteria mainly cause hypokinetic shock, while gram-positive bacteria usually cause hyperkinetic shock (Rietschel and Wagner, 2012). However, there is no relevant research to explore the prognosis of multiple Gram-negative bacteria and Gram-positive bacteria coexisting with mixed infection, as a result, there is no corresponding diagnosis and treatment as well as early-warning applications for related clinical events.

In this study, we regard that different combinations of bacterial infections will lead to different prognoses. Therefore, based on a large clinical database, we divide patients into several subgroups according to the type of bacteria infected by patients and then compare their disease severity and survival. We cluster the population based on unsupervised machine learning, which can more objectively classify patients without mixing subjective elements and is more scientific and repeatable. This research aims to reveal the underlying mechanism and its relationships with different infections of different bacteria, the therapeutic medicine, and the interventional strategy for rescuing sepsis. Based on the scientific proofs provided in evidence from real-world data, the Medical Information Mart for Intensive Care IV (MIMIC-IV) database, which is a publicly available clinical real-world database sourced from the Beth Israel Deaconess Medical Center (BIDMC), we assessed all blood culture-based bacteria clustering information. We have gained an updated interpretation would be given for the pathogenesis and the treatment of sepsis or sepsis-associated complications, and finally expected to provide references and novel strategies with perspectives for the prevention and management of sepsis.

## 2 Methods

### 2.1 Selection of data sources and patients

All the data was obtained from the Medical Information Mart for Intensive Care IV 2.0 (MIMIC-IV 2.0) critical care data set. MIMIC-IV is a relational database containing real hospital stays which includes the data of 3,82,278 patients who were admitted to the ICU of Beth Israel Deaconess Medical Center in Boston, Massachusetts between 2008 and 2019. The author Shaokang Xu (ID: 10497372) has finished the Collaborative Institutional Training Initiative (CITI) program course named “Data or Specimens Only Research” and achieved access to the database.

The selection criteria/classifications are all according to The Third International Consensus Definitions for Sepsis and Septic Shock (Sepsis-3) which is generated by the joint task force of the Society of Critical Care Medicine (SCCM) and the European Society of Intensive Care Medicine (ESICM), we extracted all the sepsis patients in the database (SOFA score ≥ 2, And there is infected ICD code according to ICD code = 99591,99592,78,552), then all patients whose blood bacterial infection record was empty were excluded, and a total of 2,339 patients were included in our study.

### 2.2 Data analytics and machine learning process

#### 2.2.1 Statistics of bacterial infection in each group under different classification conditions

In order to show the situation of patients infected with bacteria under different conditions, we classified the population according to multiple indicators, and then showed the types of susceptible bacteria under various conditions. The indicators of classification include age, sex, race, SIRS score and GCS score. Next, count the proportion of patients with positive bacterial cultures in this subgroup. The analysis process and picture drawing are completed in R Studio, and R package ggplot2 [3.3.3] is used to draw pictures.

#### 2.2.2 Unsupervised cluster analysis of population-based on bacterial infection

We use the K-means cluster mechanism learning method to cluster data, which is a data partition method based on Euclidean distance measurement. In order to determine the number of clusters during clustering, Elbow Method is used here. The data is dimensionally reduced and clustered by the algorithm. Here, all the analysis and picture drawings are completed in Python.

#### 2.2.3 Descriptive analysis of clusters after clustering

We described the situation of each cluster after clustering, including the characteristics of the main bacterial types of infection, and compared the differences of various indicators among different clusters, including GCS score, SOFA score, SAPII score and SIRS score. One-way ANOVA Test and Kruskal-Wallis H Test are used to calculate the different significance of this variable among clusters.

#### 2.2.4 Comparison of survival outcomes of each cluster

Based on the above population clustering, we drew a 7-day survival curve and a 28-day survival curve to visualize the survival results of patients. Log-rank test is used to test the difference in survival rate between clusters. R package survivor [0.49] is used for visualization, and R package survival [3.2-10] is used for statistical analysis of survival data.

## 3 Results

### 3.1 The types of bacteria infected by patients of different age, sex and race

**FIGURE 1:**
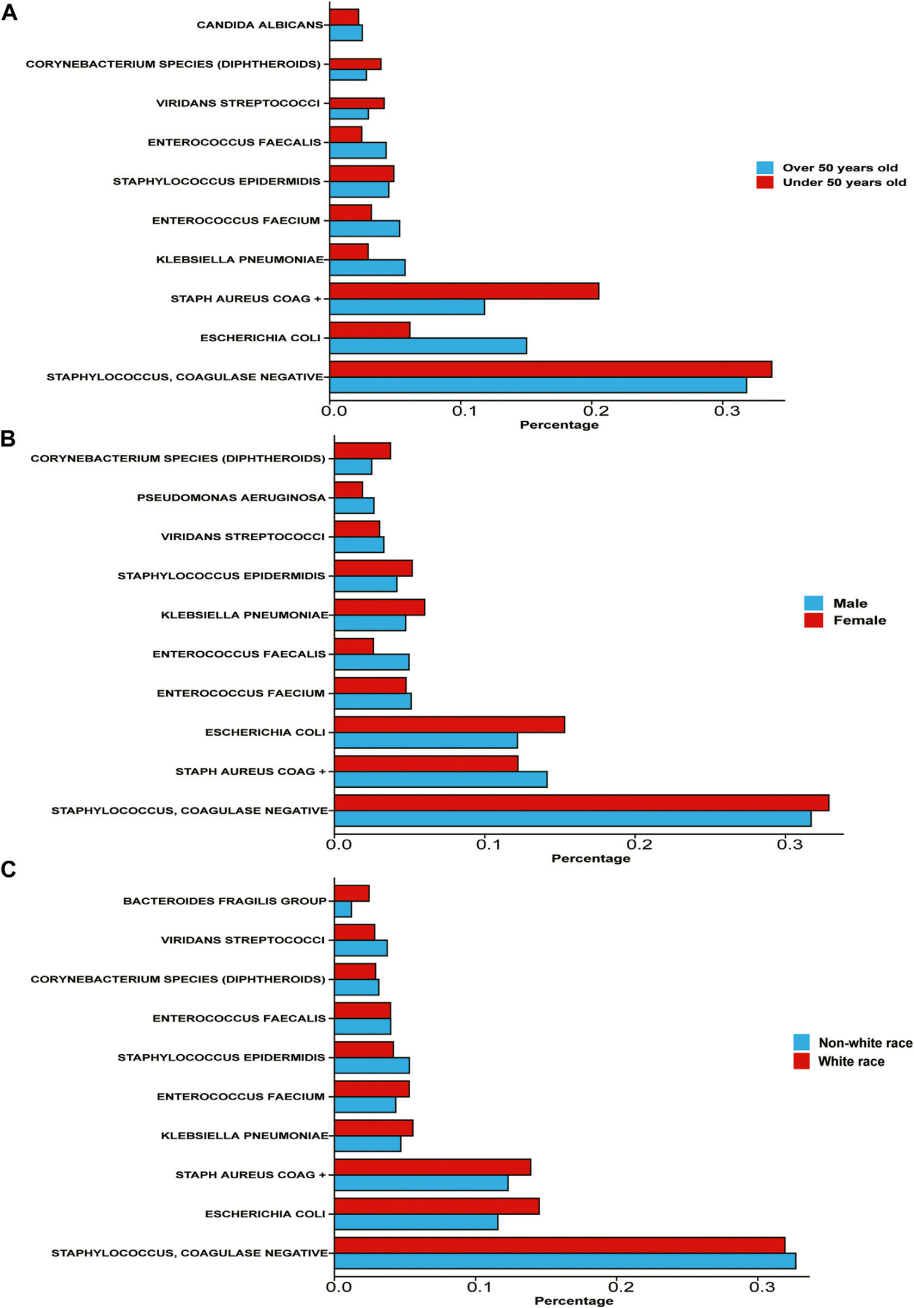
Percentage of various bacterial infections among different subgroups of population **(A)** the population is divided into two subgroups: over 50 years old and under 50 years old According to the age of patients. **(B)** The population is divided into two subgroups according to patient gender. **(C)** The population is divided into white race and non-white race according to patient race/ethnicity.

We compared the proportion of infection among different populations (Figure 1). We found that compared with people over 50 years old, people under 50 years old are more likely to be infected with *Staphylococcus* and Coagulase-negative Staphylococci (33.74% under 50 years old, 31.81% above 50 years old), but the opposite is true of *E. coli* (6.11% under 50 years old, 15.03% above 50 years old). The infection level of *S. aureus* coagulase positive (Staph Aureus COAG^+^) is also more common in people under 50 years old. Some relatively rare bacterial types, such as *K. pneumoniae*, and *Enterococcus faecium*, are also more common among people over 50 years old. In summary, gram-negative bacteria and opportunistic infection bacteria are more common in elderly patients.

However, the proportion of male and female patients infected with all kinds of bacteria is not significantly different. Among male patients, the infection rate of *Staphylococcus* and Coagulase-negative Staphylococci was 31.71%, the infection rate of Staph Aureus COAG^+^ was 14.14%, and the infection rate of *E. coli* was 12.17%. Among female patients, the infection rates of these three bacteria were 32.89%, 12.20%, and 15.31% respectively. In general, there is no significant difference between male and female patients in the type of bacterial infection.

The situation of white and non-white race (including Africans, Asians, Native Americans, Polynesians, and Alaskan natives) is more interesting. The infection rates of *Staphylococcus* and Coagulase-negative Staphylococci were not significantly different between the two populations, but the infection rates of *E. coli* in white patients were 14.50% and 11.58%, respectively. The infection rate of Staph Aureus COAG^+^ in white patients was 13.90%, and that in non-white patients was 12.30%.

### 3.2 Patients with different SIRS values and GCS scores (the first day of stay in ICU)

**FIGURE 2:**
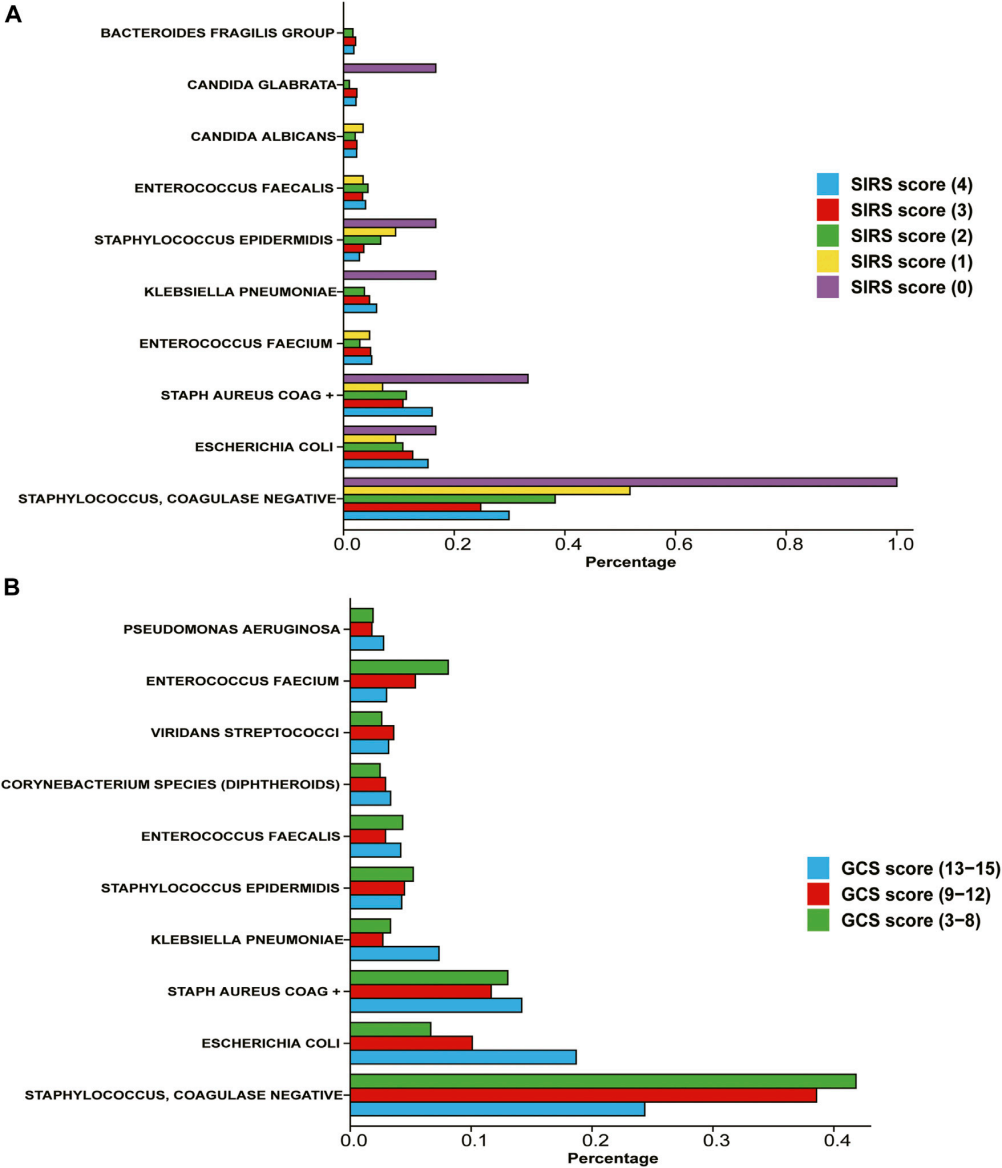
Percentage of different types of bacteria in patients with different severity **(A)** Divide the population according to the SIRS score on the first day after admission to the ICU. **(B)** Divide the population according to the GCS score on the first day after admission to the ICU.

We evaluated and counted the SIRS score and GCS score of patients on the first day after entering the ICU, and divided the population according to this standard, and counted the proportion of various bacterial infections in each scoring population. According to the WHO standard, the GCS score is classified as mild on a score of 3-8, moderate on a score of 9–12, and severe on a score of 13–15. According to the statistical results (Figure 2), we found that among the patients with high GCS score and SIRS score, the probability of infection with *Staphylococcus* and Coagulase-negative Staphylococci is relatively low, while the probability of infection with *E. coli* is relatively high.

### 3.3 Divide patients into 5 clusters by K-means clustering based on bacterial infection

Bacterial infection is often complex rather than single. And there may be synergy or inhibition between different bacteria. Therefore, the assessment of the impact of bacteria on patients cannot be simply based on a single bacterium. Based on the results of bacterial culture in all patients’ blood, we conducted unsupervised K-means machine learning cluster analysis for different patients to better classify and classify patients, so as to compare the prognosis of different types of bacterial infection. First of all, we determined that the number of clusters most suitable for clustering is 5 through Elbow Method, that is to say, it is best to divide the population into 5 types (Supplementary Figure S1). Based on this, we reduce the dimension and cluster the patient infection data (Supplementary Figure S2). The proportion of bacteria in each cluster is shown in Supplementary Material S1.

### 3.4 The severity of patients in different clusters

**FIGURE 3:**
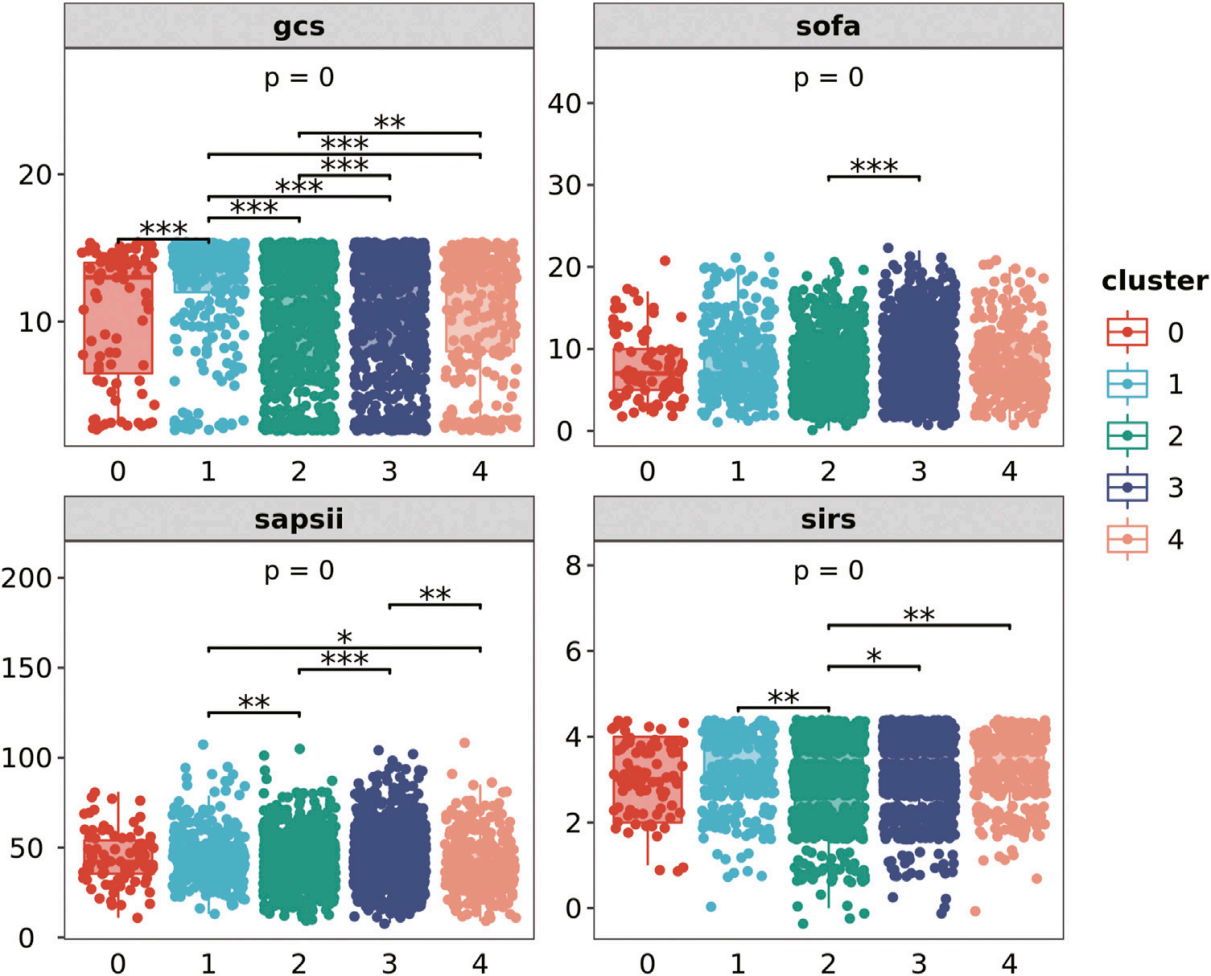
Severity of patients in each cluster on the first day.

We extract the clustered population data and compare the differences in GCS score, SOFA score, SAPII score and SIRS score (the first day) of each cluster population (Figure 3). Among the four evaluation indicators, there are differences between clusters, which have strong statistical significance. Cluster 1 has the highest GCS score, while cluster 0 and cluster 2 have relatively low scores; SOFA score is relatively balanced among all clusters. The average SAPII score of cluster 3 is lower, while the SIRS score of cluster 2 is lower. People with different types of infections have different levels of adverse symptoms.

The severity of the patient’s disease was evaluated from multiple dimensions by GCS score, SOFA score, SAPSII score and SIRS score on the first day. The *p*-values of the results of ANOVA and the *p*-values between clusters are shown in the figure.

### 3.5 The 7-day/28-day survival rate of patients in different clusters is different

**FIGURE 4:**
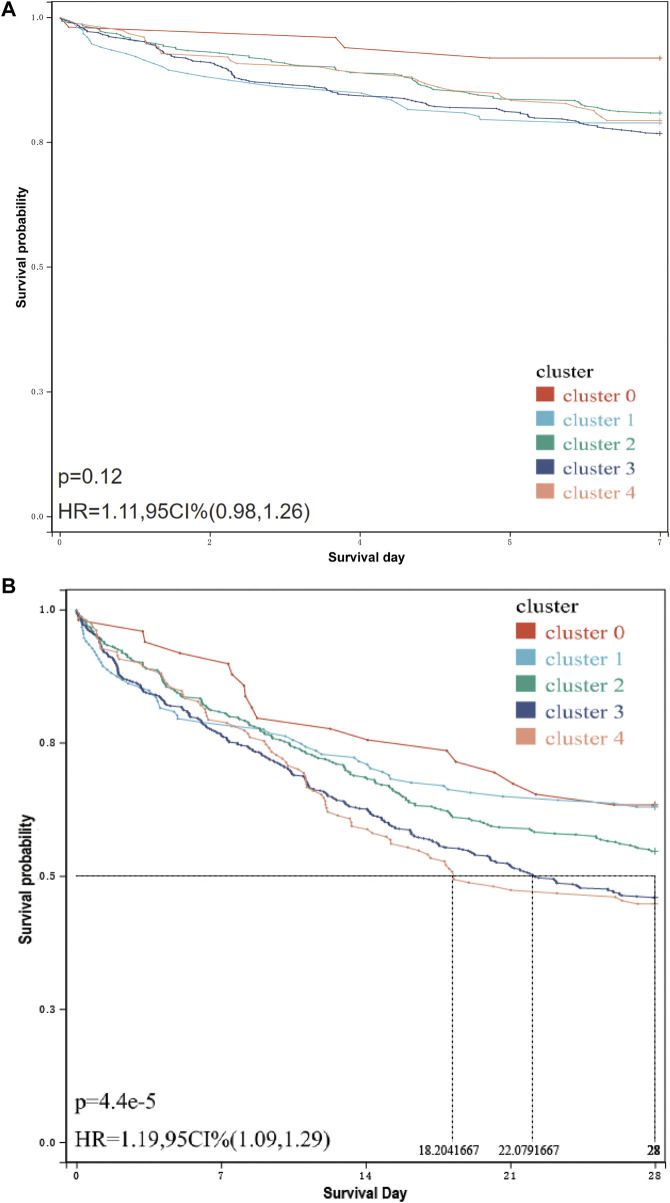
7-day/28-day survival curve of patients in each cluster. **(A)** 7-day survival curve of patients in each cluster. The related data on hazard ratio has been shown in the figure. The result of the log-rank test is *p* = 0.12, and there is no significant difference. **(B)** 28-day survival curve of patients in each cluster. The median survival time and hazard ratio have been shown in the figure. The log-rank test result is *p* = 4.4e-5, and there is a significant difference.

The results of survival analysis (Figure 4A) showed that the 28-day survival rate of cluster 3 and cluster 4 was significantly lower than that of other infection types [*p* = 4.4e-5, HR = 1.19, 95 CI% (1.09,1.29)], and the median survival time of cluster 4 was lower than that of cluster 3. However, in the 7-day survival analysis (Figure 4B), only the survival rate of cluster 3 and cluster 4 is significantly lower than that of cluster 0. For specific inter-group, log-rank, *p*-value and HR, see Supplementary Materials S2, S3.

## 4 Discussion

As a serious life-threatening disease, sepsis is defined as a life-threatening organ dysfunction, which is caused by the host’s maladjusted response to systemic infection (Jain, 2018). Although there is more and more understanding of this complex disease process, the mortality of sepsis is still the most common cause of death in non-coronary intensive care units (Mayr et al., 2014). As one of the important causes of systemic infection, the interaction between bacteria and various bacteria and the important evidence brought by them in real-world data have not been fully clarified. In this study, we conducted a retrospective study based on the large critical database MIMIC-IV 2.0. The population is divided into several subgroups based on age, sex and race, and the results of the statistical description of the proportion of various bacterial infections have surprising findings. In the elderly group, the infection rate of *Staph Aureus COAG+*, Coagulase-negative *Staphylococci* and *Staphylococcus* is relatively low, while the infection rate of *E. coli* is relatively high. In general, the infection of pathogenic *E. coli* is opportunistic and only causes infection when the human immune function is low.

Next, we divided the population into five clusters based on the results of blood bacterial culture, statistically compared the infection characteristics of the five clusters and the differences between their relevant indicators, and finally analyzed the differences in survival rates of different populations, including the 7-day survival rate and the 28-day survival rate. According to the results, we can find that the GCS score index of Cluster 1 population mainly infected by *E. coli* bacteria is significantly higher; The SAPII score index of Cluster 3 population infected by multiple bacteria is significantly lower; The 28-day survival rate of Cluster 4 population with Staph Aureus COAG + bacterial infection is significantly lower. Of course, the survival and prognosis are often associated with multiple factors, which need to be judged by combining multiple data.

In fact, different bacterial infections will lead to different outcomes. As we know, gram-negative bacteria and gram-positive bacteria are quite different in pathogenesis and subsequent treatment methods. Moreover, relevant studies have analyzed the prognosis of bacterial infections of different species and genera. However, whether the mixed infection of different bacteria will lead to different outcomes has not been studied. We believe that the synergy and competition between different bacteria will produce different pathological characteristics and clinical outcomes. Therefore, it is more meaningful to classify the population according to the situation of multiple bacterial infections and then carry out a specific treatment for this population than to use broad-spectrum drugs or only treat a certain bacterium.

In this study, all our data are from the MIMIC-IV 2.0 large critical database. We have conducted an in-depth analysis of 2,339 intensive care patients, and this data volume can be said to be quite large. It is also reasonable to use cluster analysis to divide the population. Of course, our research still has deficiencies. Our research is only limited to comparing the prognosis of patients in different clusters but does not compare and analyze the curative effect of the specific treatment methods, medication and other data of patients in a certain cluster, which is also our next research goal. In addition, our study lacks relevant data records of minors under the age of 18, because some relevant indicators and scores of minors are different from those of adults, and cannot be compared simply. Of course, there still have space for further improvement such as expanding the sample size and eliminating the error caused by confounding factors. In addition, if the study is to be truly applied to clinical practice, further improvement of the model is crucial. For example, accurate measurement of the proportion of various bacterial content, bacterial infection at a specific time point, and the sequence of infection and other factors. In addition, the model should further consider the patient’s situation, such as the change in the basic medical history of different patients and other relevant indicators. These all depend on the improvement of larger and more complete databases and algorithms.

The prevention and treatment of sepsis have always been a thorny clinical problem, especially its early diagnosis warning based on infection. It is particularly critical to make a preliminary judgment on the prognosis of patients. This study starts with the initial event of sepsis, that is, infection, and divides the population into subgroups based on the type of infection bacteria, so as to carry out more detailed management of patients, which is helpful to improve or avoid bad prognosis. The prognostic assessment predicated by blood culture-based bacteria clustering in sepsis patients could bring novel strategies and perspectives on the prevention and management of sepsis, and based on the real-world data with related scientific evidence, an updated interpretation would be given by this brief research report for the pathogenesis and the treatment as well as of sepsis or sepsis-associated complications in the near future.

## Data Availability

The original contributions presented in the study are included in the article/Supplementary Material, further inquiries can be directed to the corresponding author.
