# Supplementary figures and images for: A prognostic assessment predicated by blood culture-based bacteria clustering from real-world evidence: Novel strategies and perspectives on prevention and management of sepsis
Source: Front Mol Biosci. 2023 Mar 30;10:1160146. doi: 10.3389/fmolb.2023.1160146 (PMC10098072; doi:10.3389/fmolb.2023.1160146)

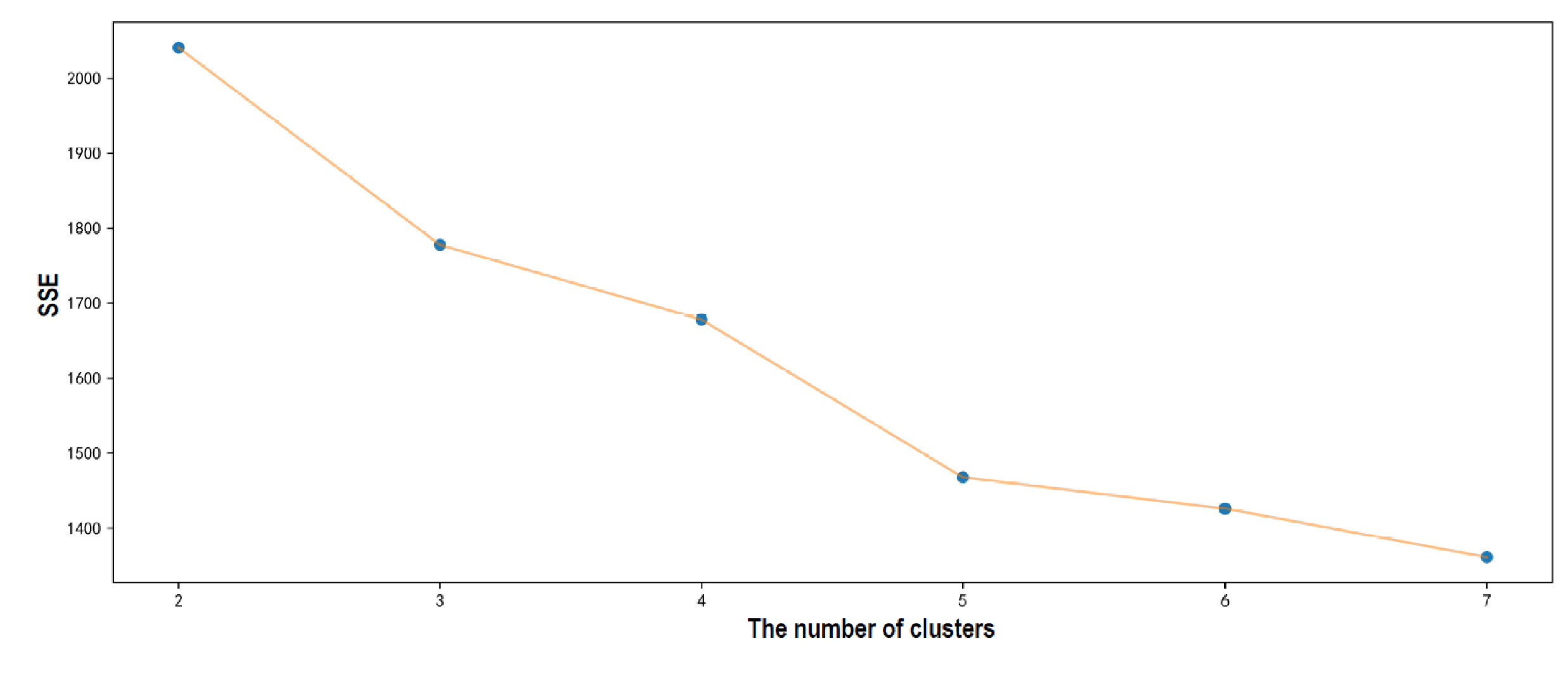

Supplement: Supplementary file 1 [file Image1.JPEG]

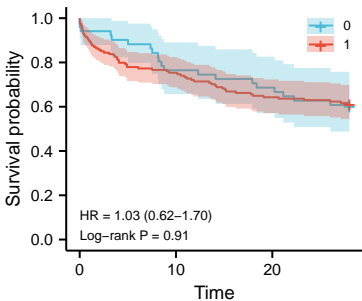

|   |     |     |    |   |
|---|-----|-----|----|---|
| 0 | 51  | 39  | 35 | 0 |
| 1 | 154 | 116 | 99 | 0 |

Supplement: Supplementary file 2 [file DataSheet1.ZIP › ╔·┤μ╟·╧▀_╢α╫Θ_01.pdf]

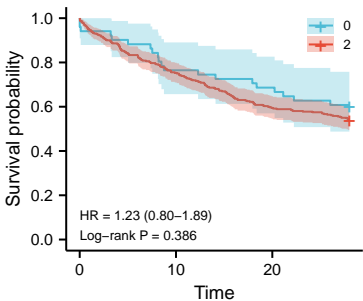

|   |     |     |     |   |
|---|-----|-----|-----|---|
| 0 | 51  | 39  | 35  | 0 |
| 2 | 360 | 271 | 214 | 0 |

Supplement: Supplementary file 2 [file DataSheet1.ZIP › ╔·┤μ╟·╧▀_╢α╫Θ_02.pdf]

Survival probability

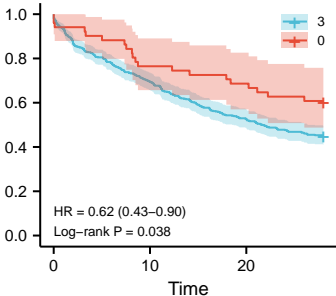

|   |     |     |     |   |
|---|-----|-----|-----|---|
| 3 | 523 | 364 | 276 | 0 |
| 0 | 51  | 39  | 35  | 0 |

Supplement: Supplementary file 2 [file DataSheet1.ZIP › ╔·┤μ╟·╧▀_╢α╫Θ_03.pdf]

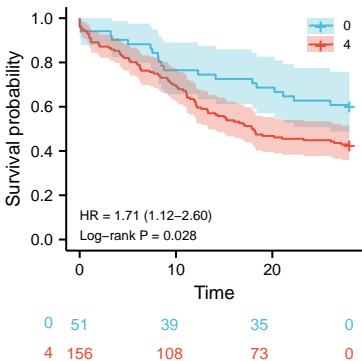

Supplement: Supplementary file 2 [file DataSheet1.ZIP › ╔·┤μ╟·╧▀_╢α╫Θ_04.pdf]

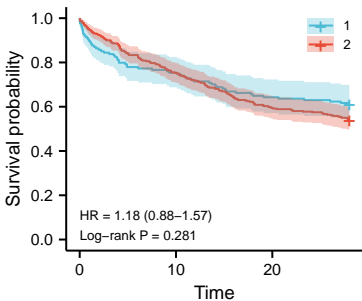

|   |     |     |     |   |
|---|-----|-----|-----|---|
| 1 | 154 | 116 | 99  | 0 |
| 2 | 360 | 271 | 214 | 0 |

Supplement: Supplementary file 2 [file DataSheet1.ZIP › ╔·┤μ╟·╧▀_╢α╫Θ_12.pdf]

Survival probability

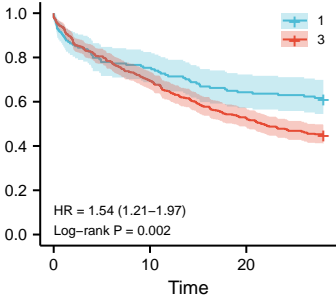

|   |     |     |     |   |
|---|-----|-----|-----|---|
| 1 | 154 | 116 | 99  | 0 |
| 3 | 523 | 364 | 276 | 0 |

Supplement: Supplementary file 2 [file DataSheet1.ZIP › ╔·┤μ╟·╧▀_╢α╫Θ_13.pdf]

Survival probability

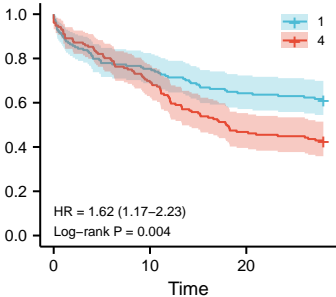

|   |     |     |    |   |
|---|-----|-----|----|---|
| 1 | 154 | 116 | 99 | 0 |
| 4 | 156 | 108 | 73 | 0 |

Supplement: Supplementary file 2 [file DataSheet1.ZIP › ╔·┤μ╟·╧▀_╢α╫Θ_14.pdf]

Survival probability

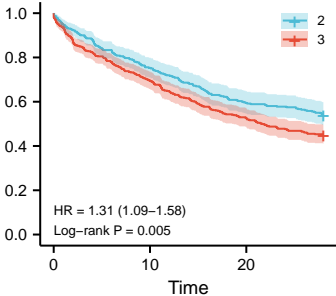

2 360

271

214

0

3 523

364

276

0

Supplement: Supplementary file 2 [file DataSheet1.ZIP › ╔·┤μ╟·╧▀_╢α╫Θ_23.pdf]

Survival probability

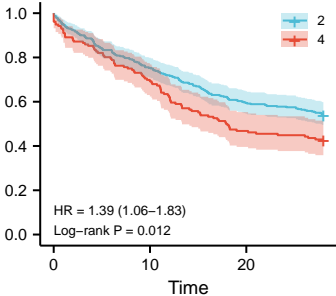

2 360

271

214

0

4 156

108

73

0

Supplement: Supplementary file 2 [file DataSheet1.ZIP › ╔·┤μ╟·╧▀_╢α╫Θ_24.pdf]

Survival probability

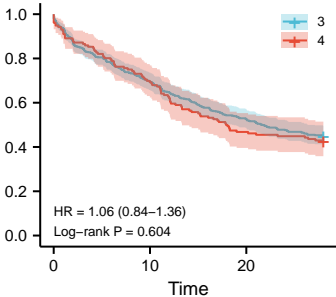

3 523

364

276

0

4 156

108

73

0

Supplement: Supplementary file 2 [file DataSheet1.ZIP › ╔·┤μ╟·╧▀_╢α╫Θ_34.pdf]

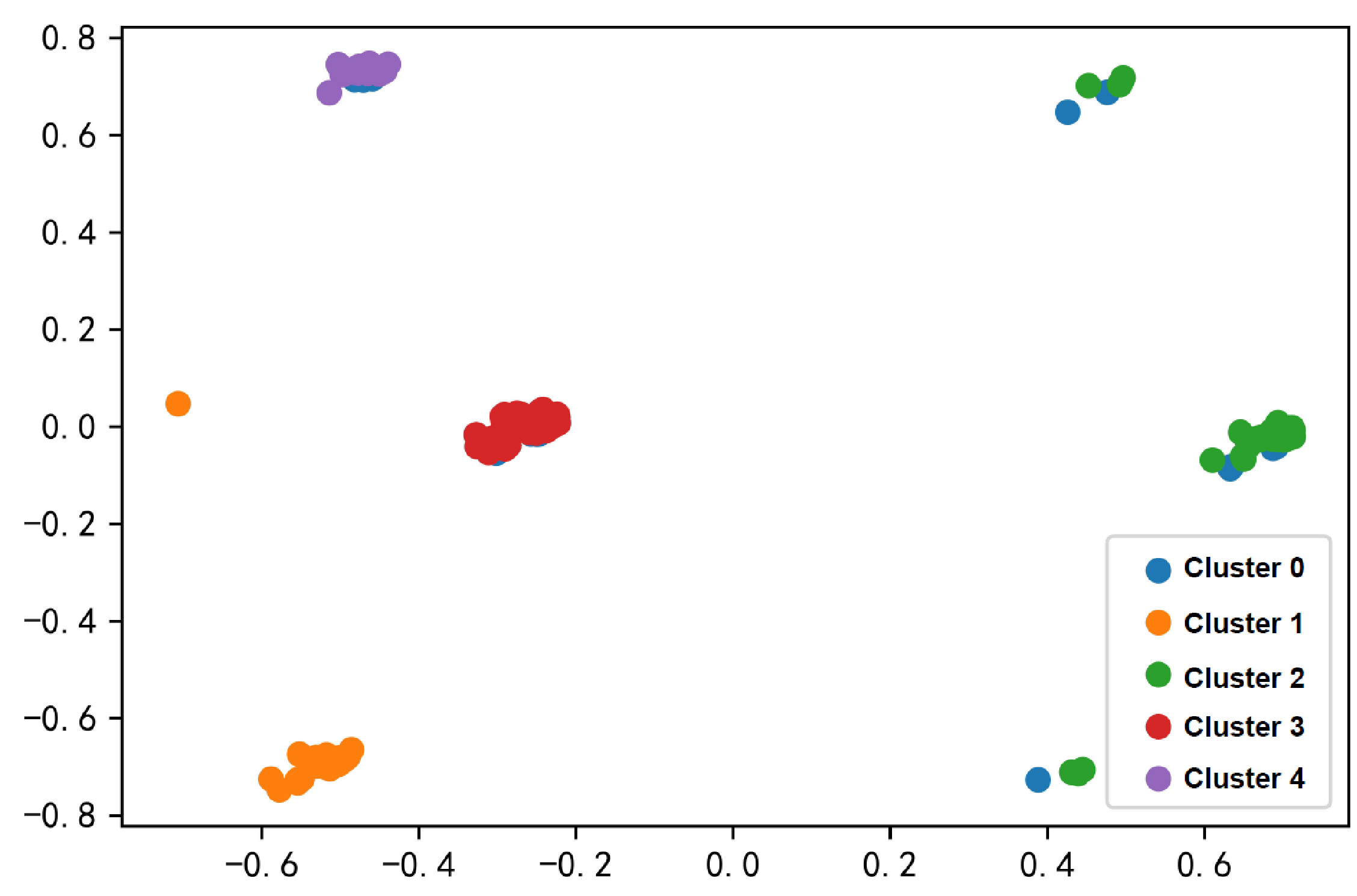

Supplement: Supplementary file 3 [file Image2.JPEG]

Survival probability

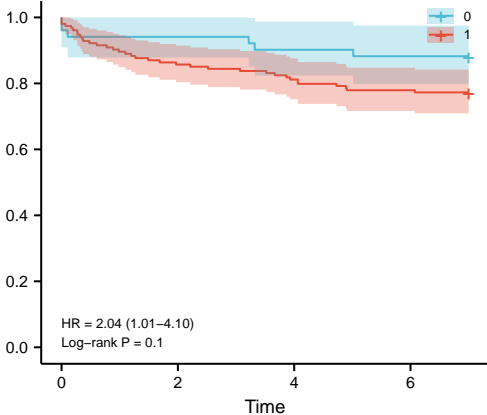

|   |     |     |     |     |   |
|---|-----|-----|-----|-----|---|
| 0 | 51  | 48  | 46  | 45  | 0 |
| 1 | 154 | 132 | 125 | 120 | 0 |

Supplement: Supplementary file 4 [file DataSheet2.ZIP › ╔·┤μ╟·╧▀_01.pdf]

Survival probability

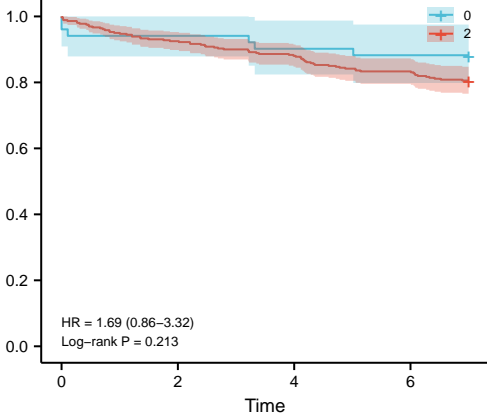

|   |     |     |     |     |   |
|---|-----|-----|-----|-----|---|
| 0 | 51  | 48  | 46  | 45  | 0 |
| 2 | 360 | 333 | 317 | 300 | 0 |

Supplement: Supplementary file 4 [file DataSheet2.ZIP › ╔·┤μ╟·╧▀_02.pdf]

Survival probability

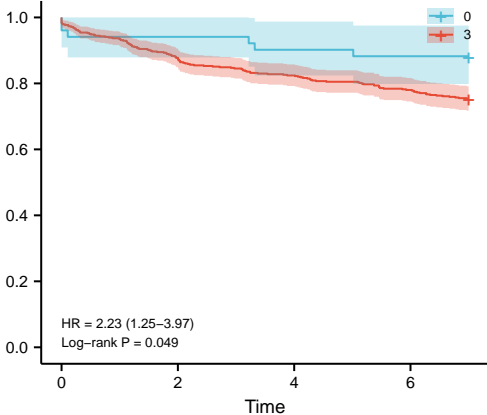

|   |     |     |     |     |   |
|---|-----|-----|-----|-----|---|
| 0 | 51  | 48  | 46  | 45  | 0 |
| 3 | 523 | 458 | 431 | 408 | 0 |

Supplement: Supplementary file 4 [file DataSheet2.ZIP › ╔·┤μ╟·╧▀_03.pdf]

Survival probability

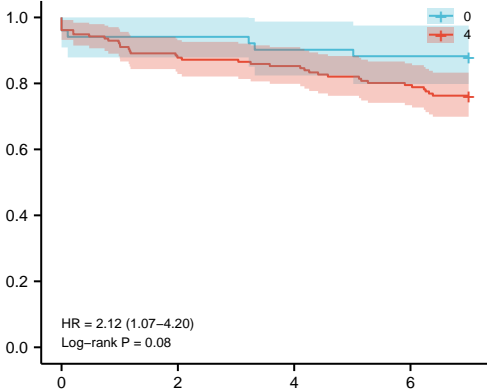

Time

|   |     |     |     |     |   |
|---|-----|-----|-----|-----|---|
| 0 | 51  | 48  | 46  | 45  | 0 |
| 4 | 156 | 137 | 133 | 124 | 0 |

Supplement: Supplementary file 4 [file DataSheet2.ZIP › ╔·┤μ╟·╧▀_04.pdf]

Survival probability

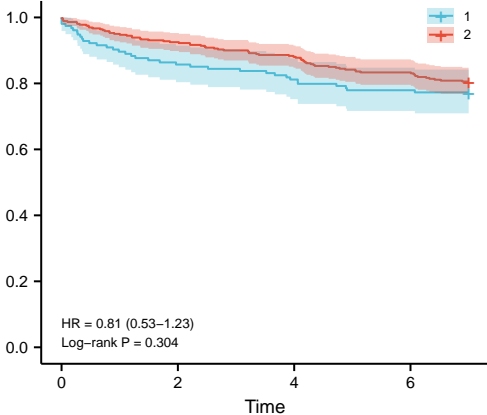

|   |     |     |     |     |   |
|---|-----|-----|-----|-----|---|
| 1 | 154 | 132 | 125 | 120 | 0 |
| 2 | 360 | 333 | 317 | 300 | 0 |

Supplement: Supplementary file 4 [file DataSheet2.ZIP › ╔·┤μ╟·╧▀_12.pdf]

Survival probability

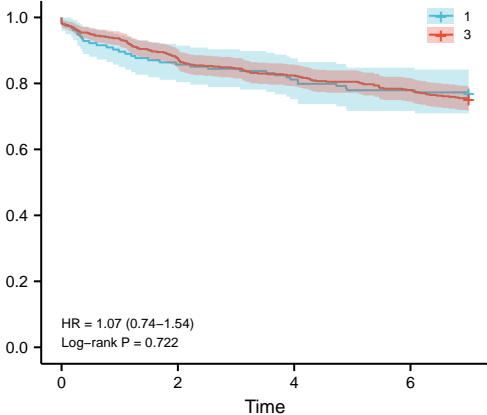

|   |     |     |     |     |   |
|---|-----|-----|-----|-----|---|
| 1 | 154 | 132 | 125 | 120 | 0 |
| 3 | 523 | 458 | 431 | 408 | 0 |

Supplement: Supplementary file 4 [file DataSheet2.ZIP › ╔·┤μ╟·╧▀_13.pdf]

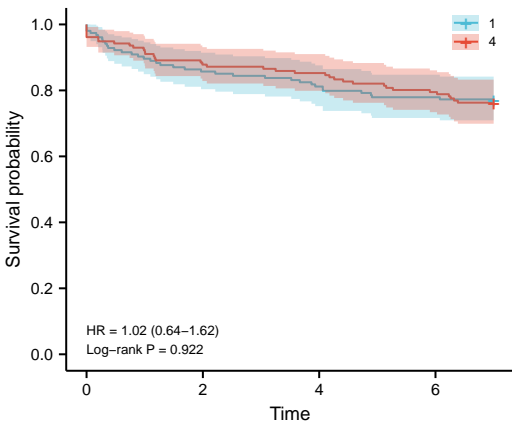

|   |     |     |     |     |   |
|---|-----|-----|-----|-----|---|
| 1 | 154 | 132 | 125 | 120 | 0 |
| 4 | 156 | 137 | 133 | 124 | 0 |

Supplement: Supplementary file 4 [file DataSheet2.ZIP › ╔·┤μ╟·╧▀_14.pdf]

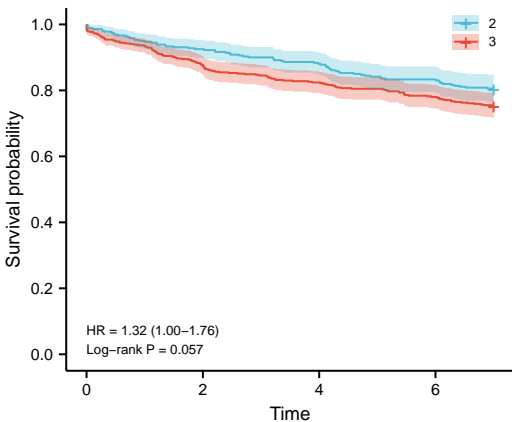

|   |     |     |     |     |   |
|---|-----|-----|-----|-----|---|
| 2 | 360 | 333 | 317 | 300 | 0 |
| 3 | 523 | 458 | 431 | 408 | 0 |

Supplement: Supplementary file 4 [file DataSheet2.ZIP › ╔·┤μ╟·╧▀_23.pdf]

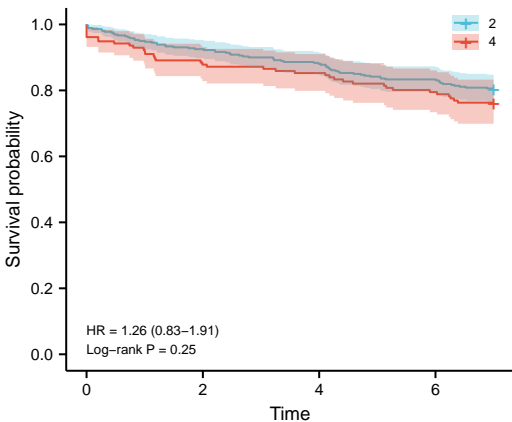

|   |     |     |     |     |   |
|---|-----|-----|-----|-----|---|
| 2 | 360 | 333 | 317 | 300 | 0 |
| 4 | 156 | 137 | 133 | 124 | 0 |

Supplement: Supplementary file 4 [file DataSheet2.ZIP › ╔·┤μ╟·╧▀_24.pdf]

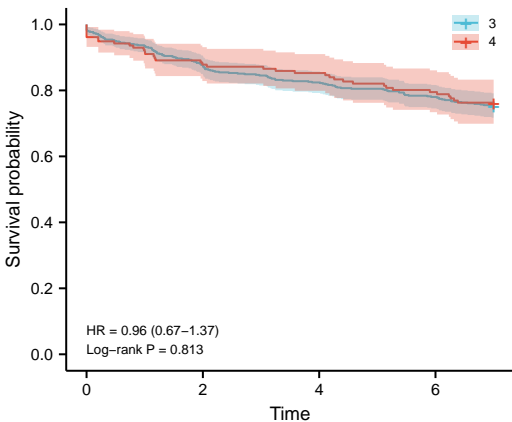

|   |     |     |     |     |   |
|---|-----|-----|-----|-----|---|
| 3 | 523 | 458 | 431 | 408 | 0 |
| 4 | 156 | 137 | 133 | 124 | 0 |

Supplement: Supplementary file 4 [file DataSheet2.ZIP › ╔·┤μ╟·╧▀_34.pdf]
